# Lycopene Antagonizes Deoxynivalenol-Induced Porcine Intestinal Epithelial Cell Senescence by Inhibiting TXNIP-Mediated NLRP3 Inflammasome Activation

**DOI:** 10.34133/research.1090

**Published:** 2026-02-06

**Authors:** Yi-Jia Song, Zi-Yan Hu, Qi Yu, Ming Lou, Yue Cheng, Ming-Shan Chen, Jia-Xin Wang, Fu-Wei Jiang, Yi-Feng Huang, Jing Zheng, Chang Liu, Zhuo-Yu Liu, Hong-Li Si, Xiao-Yi Zhang, Jin-Long Li, Yi Zhao

**Affiliations:** ^1^ College of Veterinary Medicine, Northeast Agricultural University, Harbin 150030, P.R. China.; ^2^ Key Laboratory of the Provincial Education Department of Heilongjiang for Common Animal Disease Prevention and Treatment, Northeast Agricultural University, Harbin 150030, P.R. China.; ^3^ Heilongjiang Key Laboratory for Laboratory Animals and Comparative Medicine, Northeast Agricultural University, Harbin 150030, P.R. China.

## Abstract

Mycotoxins are fungi-derived secondary metabolites that pose ecological and human health hazards. Deoxynivalenol (DON), as one of the most prevalent contaminating mycotoxins, has a detrimental impact on intestinal inflammation. Lycopene (LYC), a strong lipophilic carotenoid, is one of the most vital dietary antioxidants for human health. Thioredoxin-interacting protein (TXNIP), as a thioredoxin inhibitory protein, regulates NOD-like receptor family pyrin domain containing 3 (NLRP3) inflammasome activation. We performed this work to probe the mechanisms by which LYC antagonizes DON-induced intestinal epithelium damage and the role of TXNIP in it. In the present study, we demonstrated that LYC relieved DON-induced structural and functional injury. We observed that LYC mitigated DON-induced inhibition of cell proliferation and cell cycle arrest, thereby delaying cellular senescence. LYC also mitigated DON-induced activation of TLR4/NF-κB/TNF-α signaling and inflammatory reaction. In addition, LYC prevented DON-induced up-regulation of TXNIP, thus inhibiting NLRP3 inflammasome activation and pyroptosis. Interestingly, TXNIP overexpression reversed the protective effect of LYC on DON-induced pyroptosis and senescence, but NLRP3 inhibitor restored these impairments. Our study suggested that LYC antagonized DON-induced intestinal epithelial cell senescence by suppressing TXNIP-mediated NLRP3 inflammasome activation. These findings show that TXNIP modulates intestinal function and thereby is a new curative molecule for intestinal diseases.

## Introduction

Mycotoxins are secondary metabolites produced by molds that are regarded as the most common contaminants, contributing to adverse effects on humans and raising increasing concerns for public health worldwide [1]. A report from the Food and Agriculture Organization has estimated that a quarter of global food products are contaminated with mycotoxins [2], revealing their widespread prevalence and potential threat to food safety. Among them, deoxynivalenol (DON) is a prevalent and important mycotoxin generated by *Fusarium* species, which is frequently present in several popular cereal grains such as corn and wheat [3]. A recent global survey has revealed that the DON contamination rate exceeds 60% in cereal crops, with particularly high detection in wheat and rice [4]. A study showed that DON was present in 56.4% of cereal-based food products, with detection rates as high as 92.6% in corn and 74.2% in wheat flour [5]. In addition, DON was classified as a Group 3 carcinogen because of its toxicological properties [6]. DON is characterized by high chemical stability and heat resistance, which contribute to survival during food processing and remain in cereal-based products and animal feed [7]. Moreover, DON may infiltrate the food chain and pose serious health risks due to its bioaccumulation. Numerous studies have demonstrated that DON can exert multiple toxic effects, including intestinal toxicity, reproductive toxicity, immunotoxicity, cytotoxicity, and hepatotoxic damage [8]. The intake of DON is linked to gastrointestinal and systemic symptoms, including nausea, vomiting, abdominal pain, diarrhea, reduced food intake, and weight loss [9]. The intestinal toxicity of DON has been extensively studied, but their molecular mechanisms are still not well understood and require more elucidation.

Lycopene (LYC), a powerful antioxidant found in dietary carotenoids, is primarily present in red vegetables or fruits [10]. Due to its extensive conjugated double bond system, LYC exhibits radical scavenging capacity, the greatest antioxidant activity, and singlet oxygen quenching ability among all carotenoids [11]. Increasing evidence have shown that LYC provides multifaceted protection by suppressing oxidative stress, modulating inflammatory signaling, preventing apoptosis, and restoring mitochondrial function [12]. Notably, emerging studies have highlighted that LYC has been identified as essential for maintaining intestinal homeostasis. It has been reported that LYC alleviated mycotoxin-induced intestinal toxicity by attenuating improved antioxidants status and preserving epithelial barrier integrity [13]. Despite numerous studies highlighting the protective roles of LYC against intestinal toxicity, the underlying mechanisms remain insufficiently elucidated.

Cellular senescence is a complex process that cannot be avoided, and its key traits exhibit cell cycle arrest, which is usually linked to a secretion pattern known as aging-related secretion phenotype [14]. Cellular senescent cells have notable changes in distinct morphology, function, metabolism, and homeostasis [15]. The senescent cell accumulation has been related to the development of age-associated diseases. A growing amount of research have indicated that senescent intestinal cells undergo marked morphological alterations and metabolic dysfunction, thereby disrupting epithelial homeostasis [16]. Senescent cells promote the initiation and progression of inflammatory bowel disease (IBD) primarily through senescence-associated secretory phenotype (SASP) to drive an increase in inflammation [17]. The NOD-like receptor family pyrin domain containing 3 (NLRP3) inflammasome is formed by a protein complex that includes ASC, NLRP3, and Caspase-1, which are responsible for regulating inflammatory response [18]. However, the overactivation of the NLRP3 inflammasome can result in excessive inflammation and aggravate intestinal damage. Moreover, there is a connection between cellular senescence and the NLRP3 inflammasome [19]. NLRP3 inflammasome serves as a crucial proinflammatory signaling complex that activates Caspase-1, which, in turn, results in the release of pro-inflammatory cytokines and facilitates the onset and maintenance of senescence [20]. Some mycotoxins have been identified to induce senescence, whereas the relationship between DON exposure and senescence remains insufficiently investigated.

Pyroptosis as a form of programmed cell death is generally activated by inflammasomes and carried out by gasdermin proteins [21]. Pyroptosis is characterized by membrane pore formation, rupture, cell swelling, and lysis, which provoke strong inflammatory reactions and are involved in an extensive range of pathological processes. Growing evidence have shown that pyroptosis is closely involved in the progression of IBD. Appropriate activation of pyroptotic enhances intestinal immune defense, whereas excessive inflammasome stimulation initiates uncontrolled inflammatory cascades, thereby aggravating intestinal injury [22]. Thioredoxin-interacting protein (TXNIP) as an endogenous inhibitor of thioredoxin is essential for preserving cellular redox balance [23]. Previous findings have suggested that TXNIP aggravates cellular oxidative stress while simultaneously promoting NLRP3 inflammasome-dependent inflammatory signaling [24]. Moreover, TXNIP promotes the NLRP3 inflammasome, prompts cell apoptosis through the initiation of mitochondrial pathways, and induces pyroptosis driven by inflammatory pathways [25]. Notably, activation of the TXNIP–NLRP3 signaling axis has been reported to result in GSDMD-mediated pyroptosis, which may underlie the inflammatory damage [26]. TXNIP is increasingly recognized as a crucial regulator in maintaining intestinal balance and is linked to intestinal inflammation. Available evidence has indicated that dysregulated TXNIP expression contributes to the development of multiple intestinal disorders, such as colorectal cancer, colitis, and epithelial barrier dysfunction [27,28]. Emerging studies have further demonstrated that suppressing TXNIP activity can prevent NLRP3 inflammasome accumulation and attenuate intestinal inflammation, which are beneficial for easing intestinal diseases [29]. Although a series of evidence proves that TXNIP plays a critical role in intestinal homeostasis and disease, its potential involvement in the mechanism by which LYC relieves DON-induced intestinal injury is not clearly elucidated.

The intestinal epithelium as an active barrier between the external and internal environments protects the organism from toxins as well as maintaining intestinal homeostasis [30]. Moreover, the intestines are primarily targeted by DON. Substantial evidence has demonstrated that DON induces intestinal toxicity by impairing epithelial integrity, weakening barrier function, and altering microbial ecology, and provokes inflammatory responses, collectively contributing to the development of intestinal diseases [31]. Pigs are regarded as highly susceptible to DON toxicity, and porcine intestinal epithelial cell lines (IPEC-J2 cells), derived from newborn piglets, are commonly employed as an optimal in vitro model for investigating intestinal function. Previous studies have shown that LYC contributed to a decrease in pro-inflammatory mediators, through regulation of the TNF-α/NF-κB signaling [32]. Therefore, we utilized IPEC-J2 cells as an in vitro model to investigate how LYC modulates DON-induced alterations in intestinal function and to clarify the underlying mechanisms. TXNIP serves as a pivotal upstream regulator of NLRP3 inflammasome activation and is involved in inflammatory responses. Nonetheless, the role of TXNIP in intestinal injury after DON exposure is not fully elucidated. The objective of this work is to delineate the function and mechanistic pathways of TXNIP in DON-induced intestinal injury, thereby providing a new insight into safeguarding public health.

## Results

### LYC alleviated the DON-induced inflammatory response in IPEC-J2 cells

**Fig. 1.**
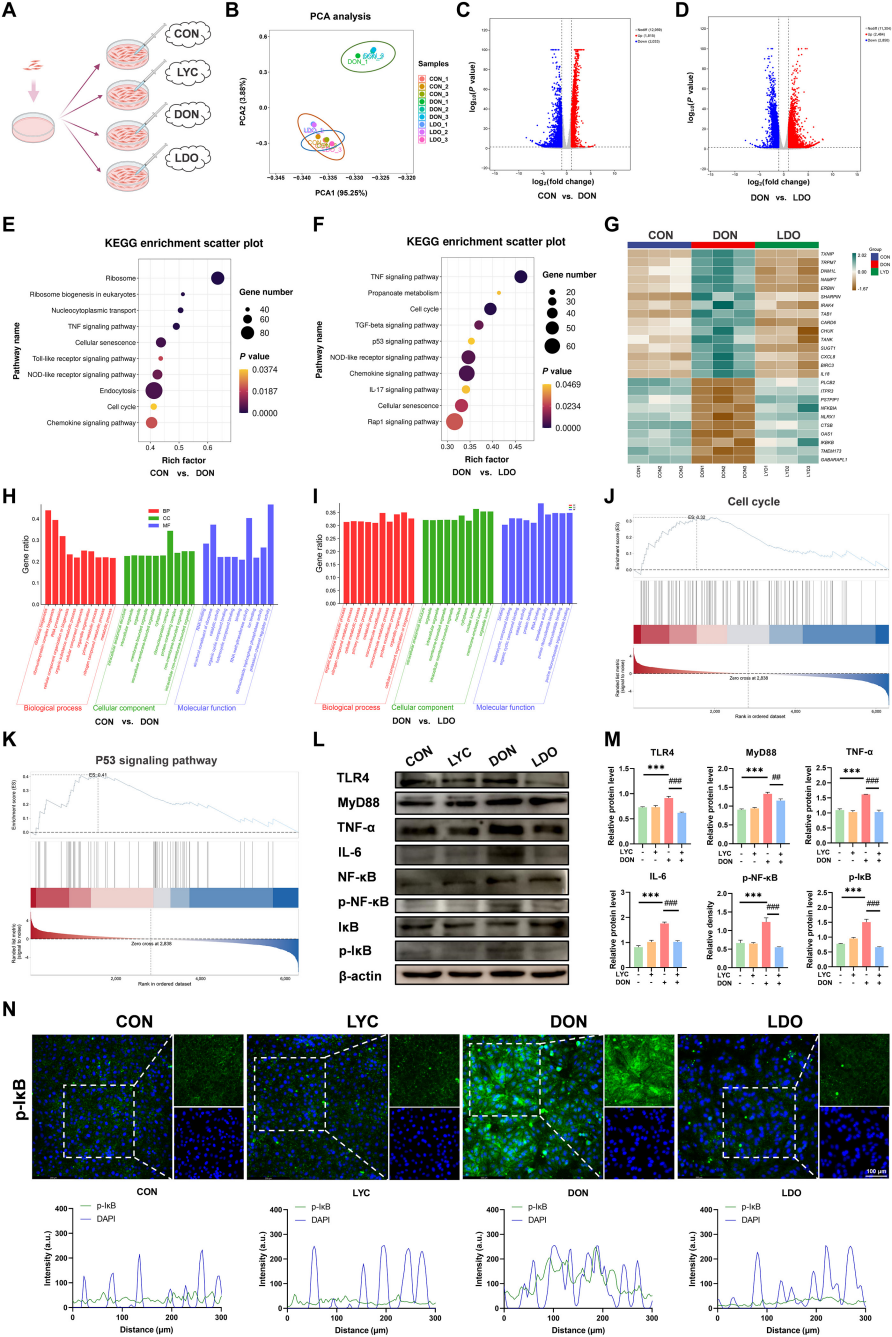
LYC alleviates the DON-induced inflammatory response in the IPEC-J2 cells. (A) The IPEC-J2 cells were treated with CON, LYC, DON, and LDO. (B) PCA. (C) Volcano plot analysis. (D) Volcano plot analysis. (E) KEGG analysis. (F) KEGG analysis. (G) Heatmap analysis. (H) GO analysis. (I) GO analysis. (J) GSEA analysis. (K) GSEA analysis. (L) Expression of inflammation-related proteins. (M) The relative level of TLR4, MyD88, TNF-α, IL-6, p-NF-κB, and p-IκB proteins. (N) Representative IF images of p-IκB. Data are presented as the mean ± SD. Symbol for the significance of differences between the CON group and DON group: ***P < 0.001. Symbol for the significance of differences between the DON group and LDO group: ^##^*P* < 0.01, ^###^*P* < 0.001.

To explore how LYC protects against DON-induced intestinal epithelial damage and clarify the underlying mechanism, an in vitro cell model was performed by exposing IPEC-J2 cells to DON and/or LYC (Fig. 1A). RNA sequencing was first conducted to study the profiles of gene expression among the CON, DON, and LYC + DON cotreatment (LDO) groups in IPEC-J2 cells. The volcano plot represents the results of the differentially expressed genes (Fig. 1C and D). Principal component analysis (PCA) demonstrated a clear separation among these groups, indicating distinct transcriptional alterations following DON exposure and LYC intervention (Fig. 1B). In addition, differentially expressed genes (DEGs) were pinpointed, followed by an analysis of Kyoto Encyclopedia of Genes and Genomes (KEGG), Gene Ontology (GO) enrichment, and Gene Set Enrichment Analysis (GSEA) (Fig. 1E to K). The KEGG enrichment pathway revealed that the TNF signaling pathway, Toll-like receptor signaling pathway, NOD-like receptor signaling pathway, and chemokine signaling pathway were the most predominantly impacted pathways in the DON-treated group (Fig. 1E). Interestingly, enrichment of the TNF signaling pathway, NOD-like receptor signaling pathway, and chemokine signaling pathway was also observed in the LDO group (Fig. 1F). To verify these transcriptomic results, Western blot was carried out to evaluate protein levels in the key inflammation-related factors. Our data have shown that DON led to a marked increase in TLR4, MyD88, TNF-α, IL-6, p-NF-κB, and p-IκB protein levels, whereas LYC treatment effectively reversed these elevations (Fig. 1L and M). Consistently, immunofluorescence (IF) analysis revealed enhanced p-IκB fluorescence intensity in the DON group, which was significantly diminished after LYC supplementation (Fig. 1N). Above all, these findings indicated that LYC attenuated DON-induced inflammatory responses by suppressing the activation of pro-inflammatory signaling pathways, including TLR4–MyD88–NF-κB pathways.

### LYC attenuated DON-induced cellular senescence in the IPEC-J2 cells

**Fig. 2.**
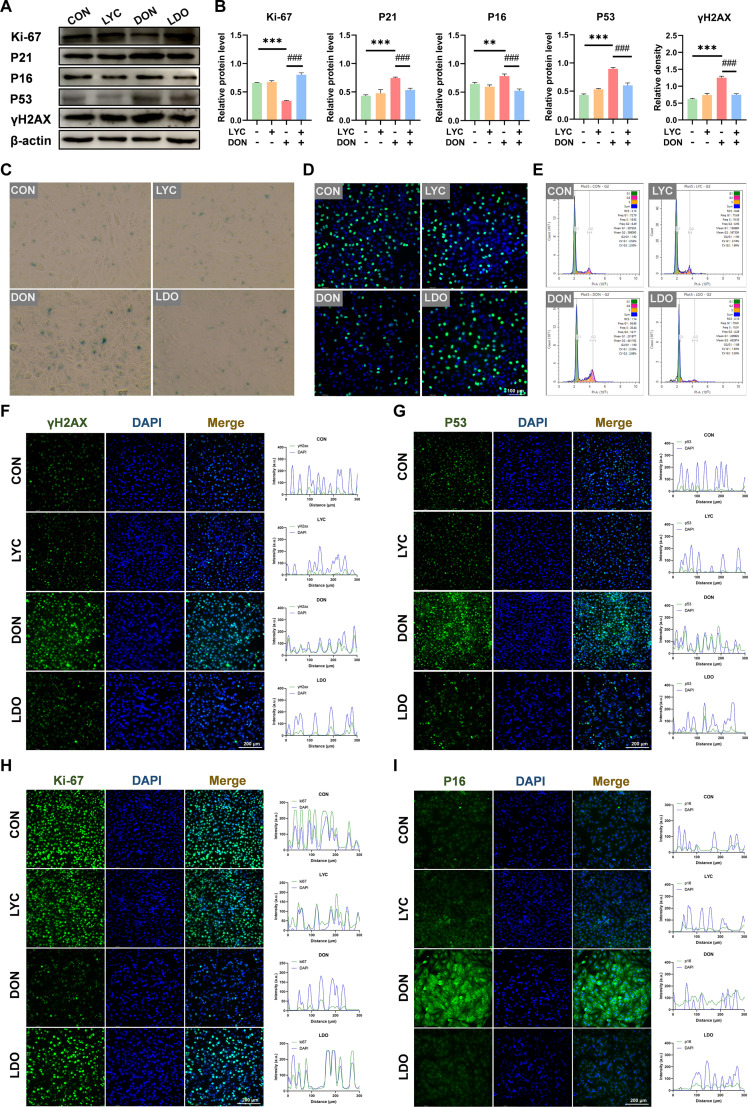
LYC attenuates DON-induced cellular senescence in the IPEC-J2 cells. (A) Expression of senescence-related proteins. (B) The relative level of Ki-67, P21, P16, P53, and γH2AX proteins. (C) SA-β-galactosidase. (D) EdU staining. (E) Flow cytometric analysis of cell cycle distribution. (F) Representative IF images of γH2AX. (G) Representative IF images of P53. (H) Representative IF images of Ki-67. (I) Representative IF images of P16. Data are presented as the mean ± SD. Symbol for the significance of differences between the CON group and DON group: **P < 0.01, ***P < 0.001. Symbol for the significance of differences between the DON group and LDO group: ^###^*P* < 0.001.

Increasing evidence indicates a close interplay between inflammation and cellular senescence, as senescent cells exhibit a SASP characterized by elevated pro-inflammatory mediators that reinforce the senescence process [33]. Consistently, KEGG analysis has indicated that DEGs were mainly involved in pathways linked to cellular senescence and cell cycle between CON and DON groups, as well as between DON and LDO groups (Fig. 1E and F). Similarly, GO enrichment analysis has suggested that DEGs were significantly associated with cellular component organization or biogenesis, and cellular component biogenesis among CON, DON, and LDO (Fig. 1H and I). Moreover, GSEA has revealed that the most obviously enriched pathways were the cell cycle and p53 signaling pathways (Fig. 1J and K). To further confirm the involvement of cellular senescence, we next estimated the key senescence-associated biomarkers to determine whether DON exposure induced cellular aging and whether LYC could reverse these effects. Western blot analysis indicated that the levels of Ki-67 were obviously down-regulated, whereas P21, P16, and P53 and γ-H2AX expression was notably up-regulated in the DON-treated group. In contrast, LYC treatment effectively prevented these DON-induced alterations (Fig. 2A and B). Consistently, IF analysis proved that fluorescence signals of P53, γ-H2AX, and P16 were increased, accompanied by the decrease of Ki-67 after DON exposure, whereas LYC administration alleviated DON-induced these changes (Fig. 2F to I). Subsequently, SA-β-gal staining determined the presence of cellular senescence. As expected, the results revealed an obvious elevation in both the proportion and number of SA-β-gal-positive cells following DON exposure, whereas LYC obviously inhibited the increase of positive cells’ ratio (Fig. 2C). In addition, 5-ethynyl-2'-deoxyuridine (EdU) incorporation assays showed that DON exposure disrupted DNA synthesis and cell proliferation, while LYC restored the ability of EdU incorporation in the IPEC-J2 cells (Fig. 2D). Flow cytometric analysis was carried out to evaluate the effect of DON and LYC on cell cycle distribution. We observed that DON induced a pronounced G2 phase arrest, while LYC treatment restored the normal cell cycle profile (Fig. 2E and Fig. S1). In general, these results demonstrate that LYC effectively mitigates DON-induced cellular senescence and restores normal proliferative capacity by suppressing inflammation in the IPEC-J2 cells.

### LYC attenuated DON-induced pyroptosis and up-regulation of TXNIP protein in the IPEC-J2 cells

**Fig. 3.**
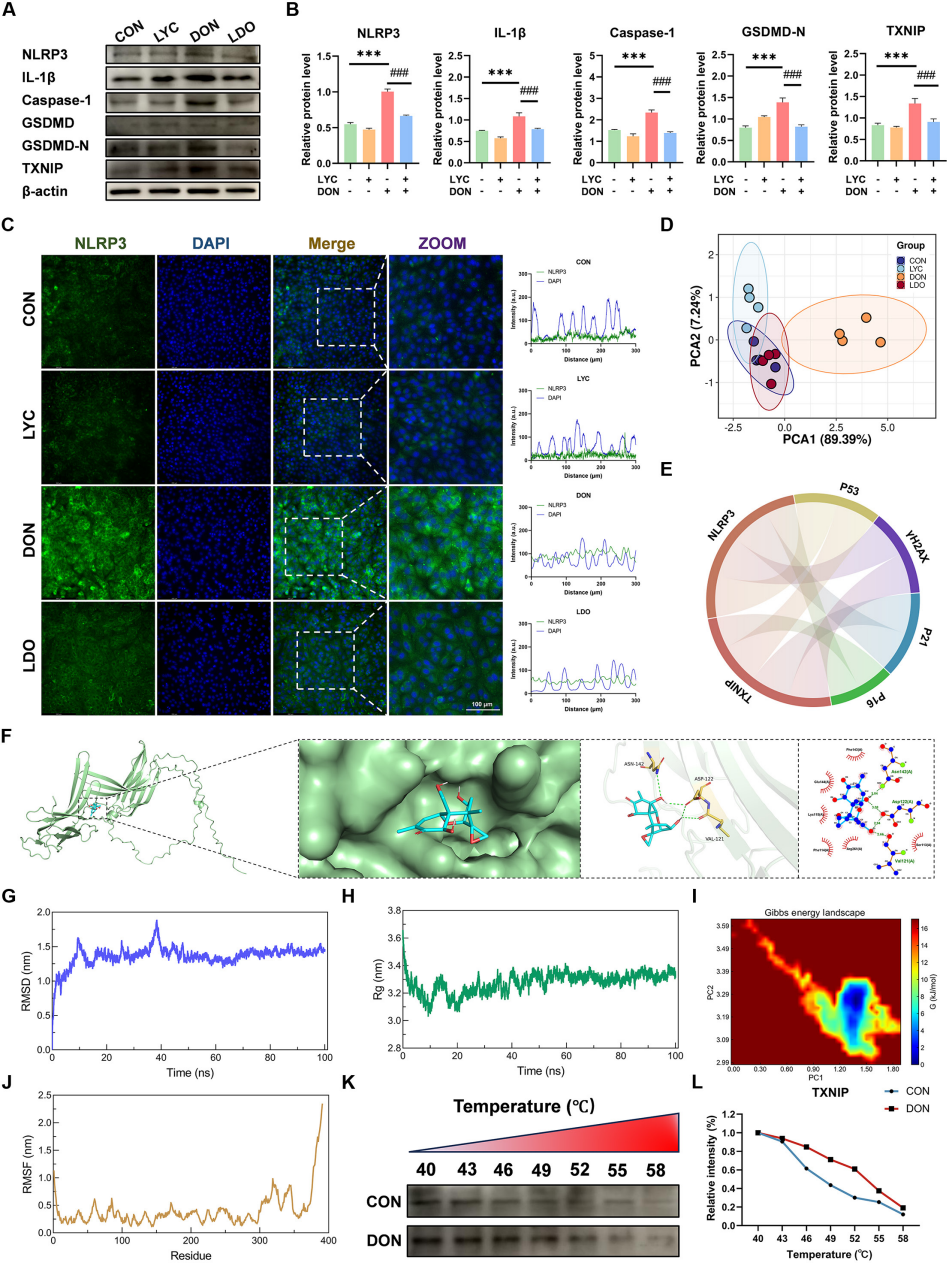
LYC attenuated DON-induced pyroptosis and up-regulation of TXNIP protein in the IPEC-J2 cells. (A) Expression of pyroptosis-related proteins. (B) The relative level of NLRP3, IL-1β, Caspase-1, GSDMD-N, and TXNIP proteins. (C) Representative IF images of NLRP3. (D) PCA. (E) Chord diagrams. (F) Molecular docking simulation for the ligand−protein binding of DON with TXNIP. (G) RMSD of the complex. (H) Rg number. (I) Gibbs free energy 2D landscape. (J) RMSF number. (K) CETSA. (L) The relative level of TXNIP intensity. Data are presented as the mean ± SD. Symbol for the significance of differences between the CON group and DON group: ***P < 0.001. Symbol for the significance of differences between the DON group and LDO group: ^###^*P* < 0.001.

Pyroptosis initiates and amplifies inflammatory signaling, thereby forming a vicious cycle of cellular damage and inflammation. To further investigate this mechanism, we assessed pyroptosis-related indicators. Western blot revealed that DON markedly elevated the levels of NLRP3, Caspase-1, IL-1β, GSDMD-N, and TXNIP, but LYC treatment effectively mitigated these elevations (Fig. 3A and B). Consistently, we found that LYC reduced the DON-induced increase of NLRP3 fluorescence (Fig. 3C). Afterward, pyroptosis-related factors were analyzed by PCA, confirming that LYC significantly alleviated the DON-induced alterations (Fig. 3D). Accumulating evidence has suggested that NLRP3 inflammasome-mediated pyroptosis plays a crucial role in age-related diseases [34]. Consistently, our results have indicated that there was a strong connection between pyroptosis and cellular senescence (Fig. 3E). As an upstream regulator of the NLRP3 inflammasome, TXNIP promotes pyroptosis and enhances inflammatory signaling, thereby accelerating the process of cellular senescence [35]. To determine whether DON directly targets TXNIP, molecular docking and molecular dynamics simulations were conducted. The results demonstrated a stable binding between DON and TXNIP (Fig. 3F), and the calculated binding energy is summarized in Table S1. Moreover, molecular dynamics simulation confirmed that the DON–TXNIP complex maintained stable structural integrity (Fig. 3G to J). Consistently, the results of cell thermal shift assay (CETSA) showed that the DON treatment markedly enhanced the thermal stability of TXNIP, further supporting the direct interaction between DON and TXNIP (Fig. 3K and L). Collectively, these findings suggest that LYC restored cellular senescence by suppressing TXNIP-mediated pyroptosis in the IPEC-J2 cells.

### TXNIP overexpression counteracted the protective effects of LYC on DON-induced inflammatory response and pyroptosis

**Fig. 4.**
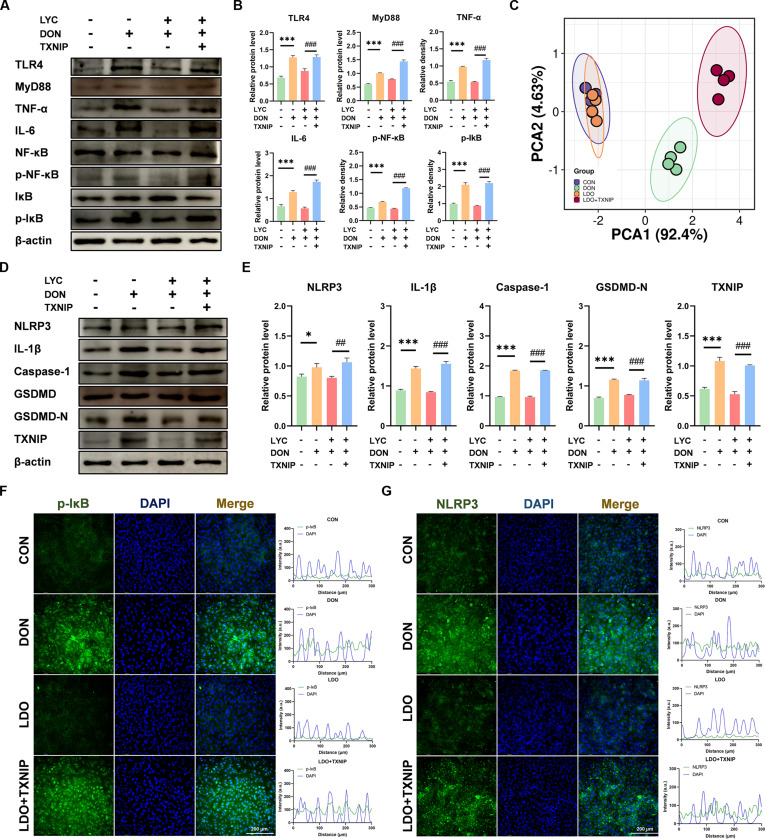
TXNIP overexpression counteracted the protective effects of LYC on DON-induced inflammatory responses and pyroptosis. (A) Expression of inflammation-related proteins. (B) The relative level of TLR4, MyD88, TNF-α, IL-6, p-NF-κB, and p-IκB proteins. (C) PCA. (D) Expression of pyroptosis-related proteins. (E) The relative level of NLRP3, IL-1β, Caspase-1, GSDMD-N, and TXNIP proteins. (F) Representative IF images of p-IκB. (G) Representative IF images of NLRP3. Data are presented as the mean ± SD. Symbol for the significance of differences between the CON group and DON group: *P < 0.05, **P < 0.01, ***P < 0.001. Symbol for the significance of differences between the LDO group and LDO + TXNIP group: ^##^*P* < 0.01, ^###^*P* < 0.001.

To investigate the potential role of TXNIP in LYC relieving DON-induced intestinal damage, TXNIP overexpression was performed in IPEC-J2 cells. TXNIP overexpression markedly up-regulated the expression of TLR4, MyD88, TNF-α, IL-6, p-NF-κB, and p-IκB in the LDO groups (Fig. 4A and B). Meanwhile, IF analysis confirmed the Western blot observations for p-IκB (Fig. 4F). Notably, LYC failed to reverse DON-induced elevation in the expression of NLRP3, IL-1β, Caspase-1, GSDMD-N, and TXNIP under TXNIP overexpression (Fig. 4D and E). IF analysis have shown the increased fluorescence intensity of NLRP3 in the DON and LDO + TXNIP groups, whereas LYC treatment effectively reduced the signal (Fig. 4G). Interestingly, PCA further proved that TXNIP overexpression impaired the restorative effects of LYC (Fig. 4C). Collectively, these findings indicate that TXNIP overexpression compromises the anti-inflammatory and anti-pyroptotic roles of LYC in DON-exposed IPEC-J2 cells.

### TXNIP overexpression attenuated the protective effects of LYC against DON-induced cellular senescence

**Fig. 5.**
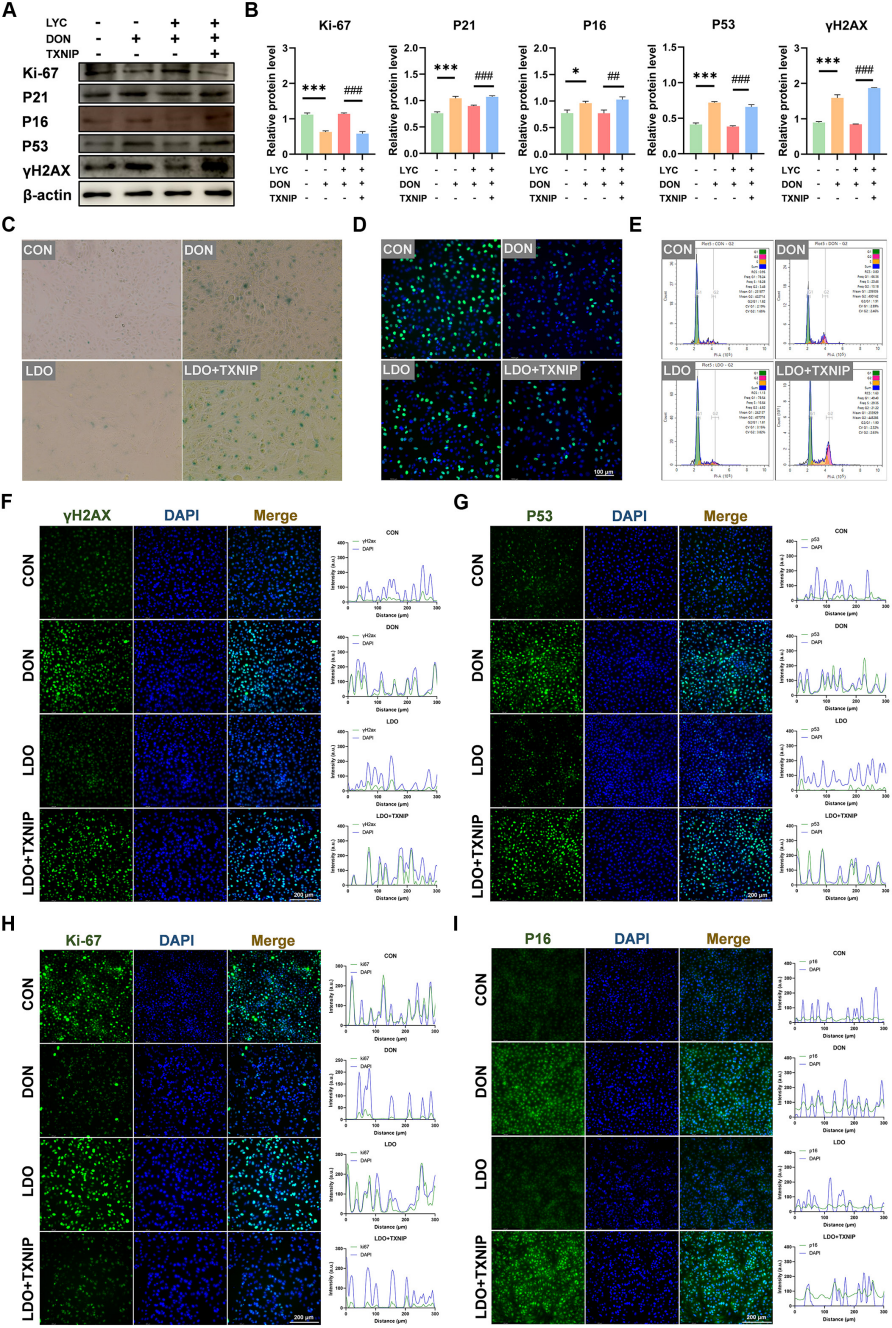
TXNIP overexpression attenuated the protective effects of LYC against DON-induced cellular senescence. (A) Expression of senescence-related proteins. (B) The relative level of Ki-67, P21, P16, P53, and γH2AX proteins. (C) SA-β-galactosidase. (D) EdU staining. (E) Flow cytometric analysis of cell cycle distribution. (F) Representative IF images of γH2AX. (G) Representative IF images of P53. (H) Representative IF images of Ki-67. (I) Representative IF images of P16. Data are presented as the mean ± SD. Symbol for the significance of differences between the CON group and DON group: *P < 0.05, **P < 0.01, ***P < 0.001. Symbol for the significance of differences between the LDO group and LDO + TXNIP group: ^##^*P* < 0.01, ^###^*P* < 0.001.

We next examined whether TXNIP overexpression affects the antagonism of LYC against DON-induced cellular senescence. Western blot analysis revealed that TXNIP overexpression markedly increased the expression of P16, P21, P53, and γ-H2AX, while reducing Ki-67 levels under DON and LYC cotreatment (Fig. 5A and B). Consistently, IF analysis showed enhanced nuclear accumulation of γ-H2AX, P16, and P53, accompanied by decreased Ki-67 fluorescence (Fig. 5F to I). Of note, we found that TXNIP overexpression elevated the proportion of SA-β-gal-positive cells after DON exposure (Fig. 5C). Furthermore, TXNIP overexpression abolished the antagonism of LYC against DON-induced suppression of cell proliferation (Fig. 5D). We also found that TXNIP overexpression reversed the LYC-mediated alleviation of DON-induced cell cycle arrest in IPEC-J2 cells (Fig. 5E and S2). Given the above, our findings proved that TXNIP overexpression weakened the antagonism of LYC against DON-induced cellular senescence.

### LYC relieved DON-induced senescence by inhibiting TXNIP-mediated NLRP3 inflammasome activation

**Fig. 6.**
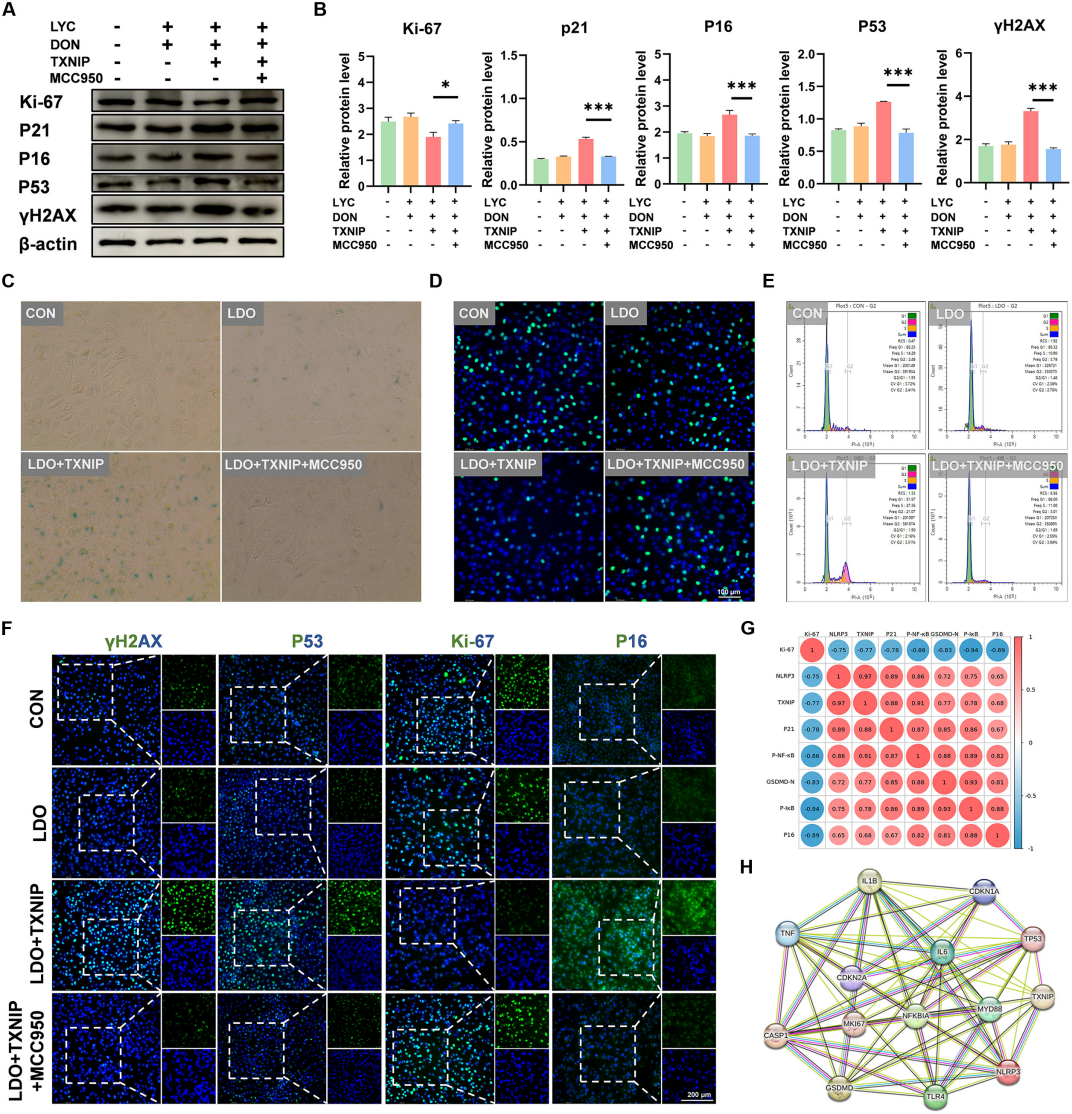
LYC relieved DON-induced senescence by inhibiting TXNIP-mediated NLRP3 inflammasome activation. (A) Expression of senescence-related proteins. (B) The relative level of Ki-67, P21, P16, P53, and γH2AX proteins. (C) SA-β-galactosidase. (D) EdU staining. (E) Flow cytometric analysis of cell cycle distribution. (F) Representative IF images of γH2AX, P53, Ki-67, and P16. (G) Correlation analysis. (H) PPI network. Data are presented as the mean ± SD. Symbol for the significance of differences between the LDO + TXNIP group and the LDO + TXNIP + MCC950: *P < 0.005, **P < 0.01, ***P < 0.001.

**Fig. 7.**
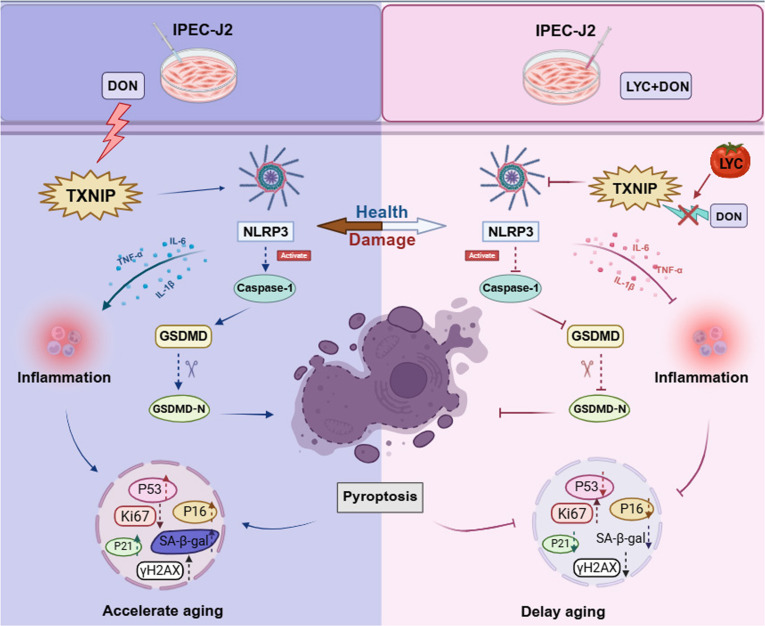
LYC antagonized DON-induced disruption of the interaction between TXNIP and NLRP3 by binding to TXNIP, inhibited NLRP3 activation and pyroptosis, and thus effectively delayed intestinal epithelium senescence. TXNIP can serve as a target for regulating mycotoxin-induced intestinal epithelium senescence and a fresh perspective into antagonizing pyroptosis.

TXNIP can interact with NLRP3 inflammasomes to activate the TXNIP/NLRP3 pathway and thus induce pyroptosis. To assess the role of the TXNIP–NLRP3 inflammasome in LYC antagonizing DON-induced intestinal epithelium damage, we validated it using an NLRP3 inhibitor MCC950. MCC950 treatment reversed the changes of Ki-67, P16, P21, P53, and γH2AX protein expression caused by TXNIP overexpression (Fig. 6A, B, and F). Moreover, MCC950 treatment led to a decrease in the percentage of positive SA-β-gal staining, suggesting that TXNIP–NLRP3 inflammasome could accelerate cellular senescence (Fig. 6C). MCC950 treatment also led to an increase in the fluorescence intensity of EdU-labeled cells, showing that the TXNIP–NLRP3 pathway could inhibit cell proliferation (Fig. 6D). We observed that MCC950 treatment inhibited a pronounced G2 phase arrest and thus restored the normal cell cycle profile (Fig. 6E and Fig. S3). Then, we performed correlation analysis and established a protein–protein interaction (PPI) network graph of related markers, which indicated the interaction among TXNIP, NLRP3, pyroptosis, and cellular senescence (Fig. 6G and H). Above all, our results showed that LYC antagonized DON-induced intestinal epithelial cellular senescence by suppressing TXNIP-mediated NLRP3 inflammasome activation (Fig. 7).

## Discussion

DON as a fungal metabolite produced by multiple *Fusarium* species is a common grain contaminant worldwide, posing a threat to public health worldwide [36]. LYC as a common non-provitamin is found in tomato [37]. Pig is susceptible to DON, especially the intestine of pig, which is the major site of DON absorption and the main target of DON attacks. However, the exact mechanisms of DON-induced disorder of porcine intestinal barrier function and the antagonism of LYC on it still remain unclear. In the present study, we proved that DON induced structural damage and dysfunction of IPEC-J2 cells. Here, we showed that DON inhibited cell proliferation, induced cell cycle arrest, and accelerated cellular senescence, which were accompanied by changes in expression of cell cycle proteins. LYC also alleviated DON-induced inflammatory reaction by activating the TLR4/NF-κB/TNF-α pathway. Moreover, LYC prevented DON-induced up-regulation of TXNIP protein expression and NLRP3 inflammasome-dependent cell pyroptosis. Of note, TXNIP overexpression reversed the antagonism of LYC on DON-induced pyroptosis and cellular senescence, but MCC950 (a NLRP3 inhibitor) reversed these impairments. This study showed that LYC antagonized DON-induced intestinal epithelium senescence by suppressing TXNIP-mediated NLRP3 inflammasome activation. These observations reveal that TXNIP may be a potential therapeutic target for treating the intestinal diseases.

Cellular senescence is a cell cycle arrest state responding to various damaging stimuli and toxic injury [38]. Excessive accumulation of senescent cells can negatively impact health and is involved in the onset and progression of age-related diseases. The cellular senescence phenotype is usually characterized by phenotypic alterations, damage response activation, and cyclin-dependent kinase inhibitor engagement. Senescent cells express and secrete cytokines, chemokines, and proteinases, which induce SASP [39]. It has been shown that mycotoxins may accelerate the development of hepatocyte aging [40]. LYC possesses distinct anti-aging actions such as combating aging indicators and alleviating chronic diseases [41]. In our work, the results showed that LYC ameliorated DON-induced changes of cellular senescence-related markers. Cell cycle arrest is a pivotal feature for the identification of senescence accompanied by various cell biological alterations. Stable cell cycle arrest suppresses apoptosis and thus promotes senescent cell accumulation during aging [42]. We found that LYC relieved DON-induced cell cycle perturbations. Numerous modifications involved in aging can be attributed to cellular senescence, which is a biological response that restricts the proliferation of damaged cells. Impaired cell proliferation is a main feature of cellular senescence [43]. Senescent cells tend to have increased levels of p16 protein expression, which suppresses cyclin-dependent kinases and results in cell cycle arrest. LYC can mediate multiple molecular mechanism disease treatment including selective anti-proliferation and apoptosis [44]. This study found that LYC could prevent DON-induced DNA damage and promote cell proliferation. These findings proved that LYC can alleviate DON-induced cellular senescence and accelerate molecular aging.

As a dynamic interface with an external environment, the intestinal epithelium needs to continuously eliminate and replace damaged cells to maintain barrier integrity. However, epithelial senescence disrupts intestinal homeostasis, leading to compromised barrier function, dysregulated immune responses, and a heightened pro-inflammatory responses [45,46]. An increasing number of studies have indicated that cellular senescence is critically involved in the pathogenesis of several intestinal diseases [17]. The accumulation of senescent cells can amplify inflammatory signaling, while the persistent release of SASP disrupts intestinal homeostasis and accelerates tissue aging [47]. Furthermore, accumulated clinical and experimental evidence have shown that oxidative stress and inflammation may lead to a range of intestinal diseases [48]. The intestinal epithelium is usually stimulated by gut microbiota and synbiotic foods, which must be presented to the immune system. Failure to balance intestinal tissue protection and repair can result in inflammatory diseases [49]. TLR4 as a protein of immune receptor signals through both MyD88-dependent pathways and plays a critical role in mediating inflammatory reactions [50]. When the TLR4/MyD88 pathway is activated, NF-κB is sequestered by IκB in the cytosol and consequently translocated to the nucleus to trigger several inflammatory cytokines, including IL-6 and TNF-α [51]. Impaired intestinal function induced by mycotoxins can result in the development of increased intestinal permeability and intestinal inflammatory diseases [52]. LYC as a potential bioactive compound for intestinal health possesses anti-inflammatory and anti-oxidant properties [53]. Consistently, we found that LYC alleviated the DON-induced activation of the TLR4/MyD88 signaling pathway and IκB-NF-κB complex, eventually causing inflammatory reactions. A strong relation between senescence and inflammation has been shown. Cellular senescence caused by inflammation is associated with aging and organ degeneration [54]. Our findings suggested that LYC relieved DON-induced inflammatory reactions by regulating the TLR4/NF-κB signaling pathway.

Among the distinct forms of cell death, pyroptosis is gaining attention in the pathogenesis of numerous diseases. Pyroptosis is often induced by inflammasomes and activated by gasdermin proteins [55]. Excessive pyroptosis can result in an immoderate inflammatory response that is involved in the development and progression of inflammatory diseases [56]. In the intestine, pyroptosis plays a primary role to maintain intestinal homeostasis. Cleaved gasdermin can induce epithelial cell death by pyroptosis and hinders restoration of intestine [57]. Gasdermin has been found to be a main factor for the repair of epithelial function and inflammation resolution [58]. A growing body of evidence indicated that mycotoxins induced pyroptosis and thus caused greater damage to the body [59]. Consistently, we found that DON induced pyroptosis and thus caused structural damage and functional impairment of IPECJ-2 cells, but LYC reversed these changes. TXNIP as a negative regulator of thioredoxin plays a key role in metabolism maintenance by activating inflammatory responses in an NLRP3 inflammasome-dependent manner, which consists of 3 components: NLRP3, ASC, and Caspase-1. Then, Caspase-1 induces the cleavage of the GSDMD-N to form lytic pores, which trigger the release of the proinflammatory cytokines and cause pyroptosis [60]. The activation of inflammasome is caused by the interaction between TXNIP and NLRP3. In this work, we found that LYC could alleviate DON-induced up-regulation of TXNIP protein expression and NLRP3 inflammasome activation. Our findings proved that LYC alleviated DON-induced disruption of the interaction between TXNIP and NLRP3 by binding to TXNIP, inhibited NLRP3 activation and pyroptosis, and thus effectively delayed intestinal epithelium senescence.

Our study confirmed that LYC antagonizes DON-induced intestinal epithelial senescence by inhibiting TXNIP-mediated cell pyroptosis. However, the study still has limitations. First, the present findings are primarily conducted using in vitro experiments based on porcine intestinal epithelial cells, and future in vivo studies, particularly porcine models, are required to validate these results. Second, ultrastructural analysis using electron microscopy would provide additional morphological evidence of pyroptotic feature and should be conducted in future investigations. Third, more comprehensive genetic manipulation strategies, including gain- and loss-of-function experiments, will be applied to future studies to further confirm the role of TXNIP.

In conclusion, our results suggested that LYC antagonized DON-induced intestinal epithelium senescence and inflammasome response of IPEC-J2. Mechanistically, LYC targeted the TXNIP, inhibiting NLRP3 inflammasome activation, which thereby alleviated DON-induced intestinal epithelium senescence. Notably, TXNIP overexpression abolished the protective effect of LYC, whereas inhibition of NLRP3 restored these impairments. Our study confirmed that TXNIP as a critical regulator involved DON-induced pyroptosis and senescence. Based on our findings, TXNIP was identified as a pivotal mediator of DON-induced intestinal epithelial injury, promoting NLRP3-driven pyroptosis and senescence. Overall, the observation that an inflammatory protein, TXNIP, serves a pivotal role in connecting senescence to pyroptosis mediated by NLRP3 inflammasome activation offers numerous opportunities to prevent and treat intestinal diseases.

## Materials and Methods

### Cell culture and treatment

IPEC-J2 cells (Prof. Dong Na Lab., NEAU, Harbin, China) were grown in DMEM/F12 (1:1) (MeilunBio, China) that consisted of 10% fetal bovine serum (Cellmax, China) and 1% penicillin–streptomycin (Biochannel, China), and maintained in a 5% CO₂ incubator. LYC (Sigma, USA; purity ≥ 90%) was dissolved in tetrahydrofuran (THF) containing 0.025% butylated hydroxy toluene and applied to the cells at a final concentration of 1 μM. DON (NCS Testing Technology Co., Ltd., Beijing, China; purity ≥ 98%) was mixed with dimethyl sulfoxide (DMSO) with a final working concentration of 500 ng/ml. The concentration of THF and DMSO was present in the culture media at a maximum concentration of 0.1% (v/v). The groups in the experiment are as follows: (a) Control group (CON): cells were cultured in complete medium without any treatment; (b) DON group (DON): cells were incubated with 500 ng/ml DON; (c) LYC group (LYC): cells were incubated with 1 μM LYC; and (d) LYC antagonizing DON group (LDO): cells were incubated with 1 μM LYC and 500 ng/ml DON.

### RNA-seq

For RNA-seq analysis, the overall method generally consisted of RNA isolation, library preparation, and subsequent data processing [61]. The sequencing was carried out by Shanghai Paisennuo Biotechnology Co., Ltd. (Shanghai, China), and procedures are provided in the "RNA-seq analysis" section in the Supplementary Materials.

### Cell transfection

The TXNIP overexpression vector was obtained from Seven Biological Technology Co., Ltd. (Beijing, China). The overexpression of TXNIP was created through the transfection of pcDNA3.1-TXNIP. Transient transfection of IPEC-J2 cells was carried out using Lipofectamine 3000 (Invitrogen, USA).

### NLRP3 inflammasome inhibition

The NLRP3 inhibitor MCC950 (MedChemExpress, USA) was employed to inhibit the NLRP3 inflammasome at a concentration of 10 nM. In brief, IPEC-J2 cells at 60% to 70% confluence was treated with 10 μM concentration of MCC950 for 2 h to inhibit NLRP3 activation.

### Cell proliferation measurement

Cell proliferation detection was carried out using the EdU cell proliferation Assay Kit (MeilunBio, Dalian, China). Briefly, IPEC-J2 cells were incubated with EdU working solution for 2 h. Following fixation with paraformaldehyde for 30 min, the IPEC-J2 cells were exposed to Apollo reaction cocktail to detect EdU incorporation. Fluorescent signals were subsequently examined by using a fluorescence microscope (Leica, Germany).

### SA-β-gal staining

The SA-β-gal staining kit (Beyotime, China) was employed to assess the activity of cell SA-β-gal. The EVOS imaging system (Invitrogen, USA) was utilized to detect senescence through SA-β-gal staining. A comprehensive description of the protocols is presented in the "SA-β-gal staining" section in the Supplementary Materials.

### Cell cycle analysis

Cell cycle distribution was detected using flow cytometry. The IPEC-J2 cells were seeded into 6-well plates at the appropriate density and exposed to the indicated conditions. After harvest, the cells were fixed overnight in ice-cold anhydrous ethanol and were treated with a cell cycle assay kit. Subsequently, cell cycle was evaluated using a flow cytometer.

### Western blot

Total protein was isolated in radioimmunoprecipitation assay buffer solution (APExBIO, Houston, USA) supplemented with phenylmethanesulfonyl fluoride (Seven, Beijing, China) and protease inhibitor cocktail (MedChem Express, USA) following established protocols [62–64]. The protein samples were electrophoresed using sodium dodecyl sulfate polyacrylamide gel electrophoresis and then transferred to nitrocellulose membranes, which were blocked with nonfat dry milk and incubated overnight at 4 °C with the corresponding primary antibodies (GeneTex, USA; Affinity, USA; ABclonal Technology, China; Proteintech, USA; Bioss, China) (Table S2). After washing with phosphate-buffered saline (PBS), incubation was executed with the appropriate secondary antibodies (Zhongshan Jinqiao Biotechnology Co., Ltd., Beijing, China) (Table S3) at 37 °C for 1 h. The bands were obtained using an Amersham Imager (GE, Switzerland) and quantified through ImageJ software.

### IF analysis

The procedure for IF staining followed the method described earlier [65–67]. Ultimately, 4',6-diamidino-2-phenylindole (Beyotime, China) staining was applied to the cell nuclei and images were captured using fluorescence microscopy (Leica, Germany). A comprehensive description of the protocols is presented in the "Immunofluorescence (IF) analysis" section in the Supplementary Materials.

### CETSA

For the CETSA experiments, the cells were lysed in PBS containing protease inhibitors by ultrasonication, and the lysates were aliquoted. Afterward, the samples were heated at the indicated temperatures, rapidly cooled on ice, and centrifuged. The results were subjected to Western blotting to determine protein stability.

### Molecular docking and molecular dynamics simulations

Molecular docking was performed with AutoDock, followed by molecular dynamics simulations using GROMACS 2020. The docking complexes were analyzed and visualized with the PyMOL program. A comprehensive description of the protocols is presented in the "Molecular docking and Molecular Dynamics Simulations" section in the Supplementary Materials.

### Statistical analysis

Data analysis was conducted with GraphPad Prism 9. Each quantitative experiment was performed a minimum of 3 times replicates, with results expressed as the mean ± SD. Statistical analysis was calculated using Student’s *t* tests to compare differences between the 2 groups or one-way analysis of variance for multiple group comparison, followed by Tukey’s post hoc pairwise comparison. The value of *P* < 0.05 was deemed to be statistically significant.

## Data Availability

The data that support the findings of this study are available from the corresponding author upon reasonable request.
