# Supplementary material for: Lycopene Antagonizes Deoxynivalenol-Induced Porcine Intestinal Epithelial Cell Senescence by Inhibiting TXNIP-Mediated NLRP3 Inflammasome Activation
Source: Research (Wash D C). 2026 Feb 6;9:1090. doi: 10.34133/research.1090 (PMC12876571; doi:10.34133/research.1090)
Supplement: Supplementary 1 — Materials and Methods Figs. S1 to S3 Tables S1 to S3 [file research.1090.f1.docx]

**Supporting Information**

Title

Lycopene antagonizes deoxynivalenol-induced porcine intestinal epithelial cell senescence by inhibiting TXNIP-mediated NLRP3 inflammasome activation

**Authors**

Yi-Jia Song^a, 1^, Zi-Yan Hu^a, 1^, Qi Yu^a, 1^, Xin Yao^a^, Ming Lou^a^, Yue Cheng^a^, Ming-Shan Chen^a^, Jia-Xin Wang^a^, Fu-Wei Jiang^a^, Yi-Feng Huang^a^, Jing Zheng^a^, Chang Liu^a^, Zhuo-Yu Liu^a^, Hong-Li Si^a^, Xiao-Yi Zhang^a^, Jin-Long Li^a, b, c^, Yi Zhao^a, b, c,^ ^*^

**Affiliations**

^a^ College of Veterinary Medicine, Northeast Agricultural University, Harbin 150030, P.R. China

^b^ Key Laboratory of the Provincial Education Department of Heilongjiang for Common Animal Disease Prevention and Treatment, Northeast Agricultural University, Harbin 150030, P.R. China

^c^ Heilongjiang Key Laboratory for Laboratory Animals and Comparative Medicine, Northeast Agricultural University, Harbin 150030, P.R. China

^*^Corresponding author.

Yi Zhao

Address: College of Veterinary Medicine, Northeast Agricultural University, Harbin, 150030, P. R. China

Tel: +86 451 55190407; fax: +86 451 55190407. E-mail address: zhaoyi@neau.edu.cn (Y. Zhao)

^1^ These authors contributed equally to this study

1. **Supporting Figures and Tabes**


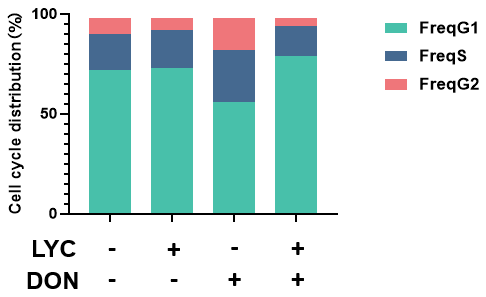


**Fig. S1.** Flow cytometric analysis of cell cycle distribution.


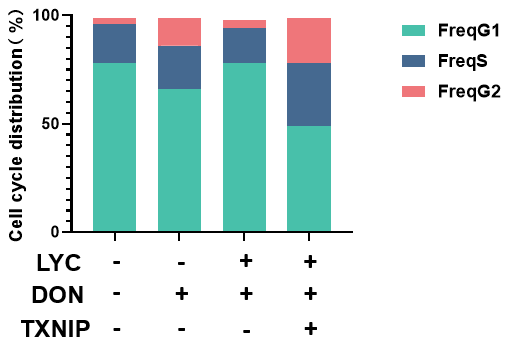


**Fig. S2.** Flow cytometric analysis of cell cycle distribution.


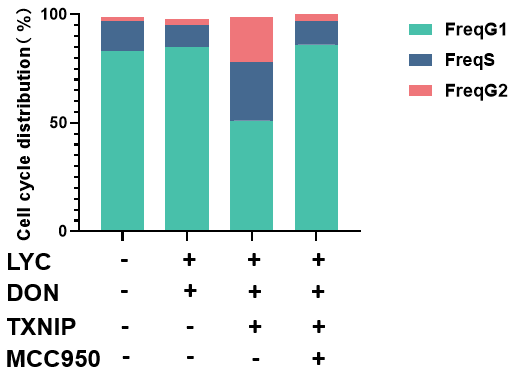


**Fig. S3.** Flow cytometric analysis of cell cycle distribution.

**Table S1.** Binding energy

| Complex | ΔVDWAALS | ΔEEL | ΔEGB | ΔESURF | ΔGGAS | ΔGSOLV | ΔTOTAL |
| --- | --- | --- | --- | --- | --- | --- | --- |
| TXNIP-  DON | -22.85  ±1.75 | -9.86  ±7.44 | 16.05  ±5.20 | -3.15  ±0.13 | -32.71  ±7.25 | 12.90  ±5.10 | -19.80  ±2.59 |

**Table S2.** **Primary antibodies obtained from different vendors in this report**

| **Target** | **Type** | **Vendor** | **Catalog no.** | **Source** | **Usage (Dilution)** | **Secondary antibody** |
| --- | --- | --- | --- | --- | --- | --- |
| β-actin | Polyclonal | GeneTex | GTX109639 | Rabbit | IB (1:2000) | Rabbit |
| NLRP3 | Polyclonal | Abclonal | A5652 | Rabbit | IB (1:1000)  IF (1:200) | Rabbit |
| Caspase-1 | Polyclonal | Abclonal | A0964 | Rabbit | IB (1:1000) | Rabbit |
| GSDMD | Polyclonal | Abclonal | A18281 | Rabbit | IB (1:500) | Rabbit |
| GSDMD-N | Polyclonal | Abclonal | A20197 | Rabbit | IB (1:500) | Rabbit |
| IL-1β | Polyclonal | Affinity | AF5103 | Rabbit | IB (1:1000) | Rabbit |
| TXNIP | Polyclonal | Abclonal | A24289 | Rabbit | IB (1:1000) | Rabbit |
| NF-κB | Polyclonal | Abclonal | A2547 | Rabbit | IB (1:1000) | Rabbit |
| p-NF-κB | Polyclonal | Abclonal | AP0124 | Rabbit | IB (1:1000) | Rabbit |
| IκB | Monoclonal | Abways | CY5026 | Rabbit | IB (1:1000) | Rabbit |
| p-IκB | Monoclonal  Polyclonal | Abways  Bioss | CY7246  bs-2513R | Rabbit  Rabbit | IB (1:1000)  IF (1:100) | Rabbit  Rabbit |
| TLR4 | Polyclonal | Bioss | bs-20594R | Rabbit | IB (1:1000) | Rabbit |
| MyD88 | Polyclonal | Affinity | AF5195 | Rabbit | IB (1:1000) | Rabbit |
| [TNF-α](http://www.baidu.com/link?url=mzyHLjExYkkUfUW4nX2Mwz66TVc3MxObv5lQ-W1WiTpQ5g6ygLzBb4V7PSrB0HRWvdClxQ16AAsCXtMbMXP2vhVM14IqVSOP8JQai5WBUGe) | Polyclonal | Abclonal | A11534 | Rabbit | IB (1:1000) | Rabbit |
| IL-6 | Polyclonal | Abclonal | A0286 | Rabbit | IB (1:1000) | Rabbit |
| Ki-67 | Polyclonal | Abclonal | A19619 | Rabbit | IB (1:1000)  IF (1:200) | Rabbit |
| P53 | Polyclonal | Bioss | bs-8687R | Rabbit | IB (1:1000)  IF (1:200) | Rabbit |
| P21 | Polyclonal | Abclonal | A1483 | Rabbit | IB (1:1000) | Rabbit |
| P16 | Polyclonal | Abclonal | A0262 | Rabbit | IB (1:1000)  IF (1:100) | Rabbit |
| γH2AX | Monoclonal  Monoclonal | STARTER  Abclonal | S-1772-179  AP0687 | Rabbit  Rabbit | IB (1:1000)  IF (1:200) | Rabbit  Rabbit |

Abbreviations used: IB – immunoblot analysis, IF – immunofluorescence microscopy.

**Table S3. Secondary antibodies obtained from different vendors in this report.**

| **Target** | **Vendor** | **Catalog no** | **Species** | **Usage (Dilution)** |
| --- | --- | --- | --- | --- |
| HRP-conjugated Goat anti-Rabbit IgG (H+L) | ZSGB-BIO | ZB-2301 | Rabbit | IB (1:3000) |
| [Goat Anti-Rabbit IgG H&L (Alexa Fluor® 488)](https://www.abcam.cn/products/secondary-antibodies/goat-rabbit-igg-hl-alexa-fluor-488-ab150077.html) (green) | abcam | ab150077 | Rabbit | IF (1:200) |

Abbreviations used: IB – immunoblot analysis, IF – immunofluorescence microscopy.

1. **Supplementary Materials and Methods**

**RNA-seq analysis**

Total RNA was isolated using a TRIzol total RNA extractibn kit (TIANGEN, Cat. No. DP424), which yielded > 2 μg of total RNA per sample. RNA quality was examined by 0.8% agarose gel electrophoresis and spectrophotometry. High-quality RNA with a 260/280 absorbance ratio of 1.8-2.2 was used for library construction and sequencing. Illumina HiSeq library construction was performed according to the manufacturer's instructions (Illumina,USA). Oligo-dT primers are used to transverse mRNA to obtain cDNA (APExBIO, Cat. No. K1159). Amplify cDNA for the synthesis of the second chain of cDNA. Purify cDNA products by magnetic beads. After library construction, library fragments were enriched by PCR amplification and selected according to a fragment size of 350-550 bp. The library was quality-assessed using an Agilent 2100 Bioanalyzer (Agilent, USA). The library was sequenced using the Illumina NovaSeq 6000 sequencing platform (Paired end150) to generate raw reads. Raw paired-end fastq reads were filtered by TrimGaloreto discard the adapters and low-quality bases via calling the Cutadapt tool. The clean reads obtained were then aligned to the mm10 mouse genome using HISAT2, followed by reference genome-guided transcriptome assembly and gene expression quantification using StringTie. Differentially expressed genes (DEGs) were identified by DEseq2 (for sample with replications) or edgeR (for sample with no replication) with a cut-off value of log2|fold-change|>1 and p-adjust <0.05. The cluster Profiler was used to perform functional enrichment analysis for the annotated significant KEGG pathway categories. Terms with p<0.05 were considered significant.

**Molecular docking and Molecular Dynamics Simulations**

PubChem and PDB were used to find the chemical and conformational information of the relevant proteins (TXNIP) and small-molecule compounds (DON). The AutoTools software was used to remove the redundant protein chains, ligands and water molecules with hydrogenation before running docking experiments. The AutoGrid software was used to calculate the energy lattice points. AutoDock Vina was used to simulate the docking condition between proteins (TXNIP) and small molecules (DON). Autogrid calculations were performed, selecting AutoDock Vina as the docking algorithm, and AutoDock was used for molecular docking. The binding energy results were visualized using PyMOL.

The resulting proteins from docking were separated from the best small molecule ligands, and small molecule force field files were generated by the antechamber tool in Ambertools software, and the small molecule force field files were converted into gromacs force field files by the acpype software tool. GAFF force field was used for small molecules, while AMBER14SB force field and TIP3P water model were used, and the files of protein and small molecule ligand were combined to construct the simulation system of the complex. Molecular dynamics simulations (MD) using Gromacs2022 program under constant temperature and periodic boundary conditions. During the MD simulation, all involved hydrogen bonds were constrained using the LINCS algorithm with an integration step size of 2 fs. The electrostatic interactions were calculated by the (Particle-mesh Ewald) PME method and the cutoff value was set to 1.2 nm. The non-bonded interaction cutoff was set to 10 Å and updated every 10 steps. The simulation temperature was controlled at 298 K using the V-rescale temperature coupling method and the Berendsen method to control the pressure at 1 bar. At 298 K, 100 ps, NVT, equilibration simulation with NPT, and 100 ns, MD simulation of the complex system, preserving the conformation every 10 ps. After the simulation, the simulated trajectories were analyzed using VMD and Pymol and MMPBSA binding free energy analysis between the protein and small molecule ligands using the g_mmpbsa program.

**SA-β-gal staining**

Cellular senescence was detected using a SA-β-gal staining kit according to the manufacturer's instructions. Briefly, after the indicated treatments, IPEC-J2 cells were washed with PBS and fixed with the provided fixative solution for 15 min at room temperature. Following fixation, the cells were then washed and incubated with the SA-β-gal staining working solution overnight at 37°C in a dry incubator. After incubation, the cells were rinsed with PBS, and images were captured using an optical microscope. Senescent cells were identified by the presence of blue cytoplasmic staining.

**Immunofluorescence (IF) analysis**

Immunofluorescence (IF) analysis was performed as previously described with minor modifications. After the indicated treatments, IPEC-J2 cells were washed with PBS and fixed with cold methanol for 15 min at room temperature. The cells were then permeabilized and blocked with a solution containing 5% normal serum and 0.3% Triton X-100 for 1 h to minimize nonspecific binding. Subsequently, the cells were incubated with the appropriate primary antibodies diluted in antibody dilution buffer overnight at 4 °C. After washing, the cells were incubated with fluorochrome-conjugated secondary antibodies for 1 hours at room temperature in the dark. Nuclei were counterstained with DAPI. Finally, images were captured fluorescence microscopy (Leica, Germany).
